# Screening of saponins and sapogenins from *Medicago* species as potential PPARγ agonists and X-ray structure of the complex PPARγ/caulophyllogenin

**DOI:** 10.1038/srep27658

**Published:** 2016-06-10

**Authors:** Roberta Montanari, Davide Capelli, Aldo Tava, Andrea Galli, Antonio Laghezza, Paolo Tortorella, Fulvio Loiodice, Giorgio Pochetti

**Affiliations:** 1 Istituto di Cristallografia, Consiglio Nazionale delle Ricerche, Montelibretti, 00015 Monterotondo Stazione, Roma, Italy; 2 Consiglio per la Ricerca in Agricoltura e l’Analisi dell’Economia Agraria (CREA-FLC), Centro di Ricerche per le Produzioni Foraggere e Lattiero Casearie, Viale Piacenza 29, 26900 Lodi, Italy; 3 Dipartimento di Farmacia-Scienze del Farmaco, Università degli Studi di Bari “Aldo Moro”, Via E. Orabona 4, 70126 Bari, Italy

## Abstract

A series of saponins and sapogenins from *Medicago* species were tested for their ability to bind and activate the nuclear receptor PPARγ by SPR experiments and transactivation assay, respectively. The SPR analysis proved to be a very powerful and fast technique for screening a large number of compounds for their affinity to PPARγ and selecting the better candidates for further studies. Based on the obtained results, the sapogenin caulophyllogenin was proved to be a partial agonist towards PPARγ and the X-ray structure of its complex with PPARγ was also solved, in order to investigate the binding mode in the ligand binding domain of the nuclear receptor. This is the first known crystal structure of a sapogenin directly interacting with PPARγ. Another compound of the series, the echinocistic acid, showed antagonist activity towards PPARγ, a property that could be useful to inhibit the adipocyte differentiation which is a typical adverse effect of PPARγ agonists. This study confirms the interest on saponins and sapogenins as a valuable natural resource exploitable in the medical and food industry for ameliorating the metabolic syndrome.

PPARγ is a crucial regulator of glucose and lipid homeostasis and an important pharmacological target for treating metabolic diseases^1^,^2^,^3^. PPARγ full agonists are strong insulin-sensitizing agents^4^. However, over-activation of PPARγ can lead to serious side effects including weight gain and steatosis, for this reason PPARγ partial agonists are more desirable^5^,^6^. On the other hand, PPARγ antagonists are also interesting targets because may inhibit lipogenesis and adipocyte differentiation, reduce fat weight and improve insulin resistance in the obesity state^7^,^8^. Obesity is also associated with a low-grade inflammation in white adipose tissue and liver, which may exacerbate insulin resistance, steatosis and diabetes. Control of inflammation seems important in the clinic treatment of the metabolic diseases^9^.

Some medicinal plants have been traditionally used to treat this kind of metabolic diseases because of their hypoglycemic and antidiabetic properties. Saponins are a class of chemical compounds found in particular abundance in various plant species which have been reported to exhibit hypoglycemic potential in diabetic states^10^,^11^, and attracted a lot of interest because of their potent, hypolipidemic and insulin-like properties^12^,^13^,^14^,^15^.

Saponins are biologically active plant-derived glycosides consisting of a sugar moiety linked to a hydrophobic aglycone (sapogenin) with a triterpenoid or a steroid structure. They may have one (monodesmosidic) or more (bi- and tridesmosidic) linear or branched sugar chains linked to the aglycone mojety through an ether or ester bond. Due to their variety of chemical structures, naturally occurring saponins display a broad diversity of polarity, hydrophobicity and acidity that determine their various biological and pharmacological features^16^.

Saponins from *Platycodi radix* have been shown to improve homeostasis in type 2 diabetic states, partly by enhancing hepatic and adipocyte insulin sensitivity which is achieved by activating PPARγ^17^. They also inhibit lipogenesis through AMPKa-PPARγ2 in 3T3-L1 cell and modulate fat accumulation in obese mice^18^. Saponins and sapogenins were recently studied for their anti-inflammatory effect due to the inhibition of NF-κB and for their effect on PPAR transcriptional activity^19^,^20^. Particularly, several oleanane-type triterpenoid saponins from the roots of *Pulsatilla koreana* inhibited TNFα-stimulated NF-κB activation in a dose-dependent manner, with IC_50_ values ranging from 0.75–8.30 μM, repressing the expression of the iNOS and ICAM-1 genes, which play important roles in the inflammatory response^21^,^22^. The same compounds also significantly activated the transcriptional activity of PPARs in a dose-dependent manner, with EC_50_ values up to 1 μM. Moreover, protopanaxatriol, a monoglucoside sapogenin present in the root of *Panax ginseng*, showed antagonist activity towards PPARγ^20^. It specifically inhibited the transactivation activity of PPARγ, but not that of PPARα, β/δ and LXR α,β, by repressing the adipocyte differentiation and ameliorating obesity, insulin resistance, steatosis and hyperlipidemia in diet-induced obesity mice.

Based on the above reported evidence, saponins and sapogenins from *Medicago* species were considered and used to study their effects on PPARγ. In the genus *Medicago*, saponins are a complex mixture of triterpenic pentacyclic glycosides with medicagenic acid, hederagenin, bayogenin, zanhic acid and soyasapogenol B as the most abundant aglycones. The most abundant monosaccharide units found in the *Medicago* saponins are: arabinose, rhamnose, xylose, glucose and glucuronic acid. These compounds have been shown to possess a broad spectrum of biological properties such as fungicidal, molluscicidal, insecticidal, nematicidal, allelopathic, antiviral, cytotoxic and hemolytic activities^23^.

In the present paper a series of previously purified and characterized saponins and sapogenins from *Medicago* species were tested for their ability to bind and activate PPARγ by SPR experiments and transactivation assay, respectively. Based on the obtained results, one of the most active compounds was also considered for further studies by resolving the X-ray structure of its complex with PPARγ, in order to investigate the binding mode in the ligand binding domain (LBD) of the nuclear receptor. ITC experiments were also performed on this compound in comparison with the fatty acid 13-(*S*)-HODE, a natural agonist of PPARγ showing a similar binding mode.

## Results

### Surface Plasmon Resonance

**Figure 1:**
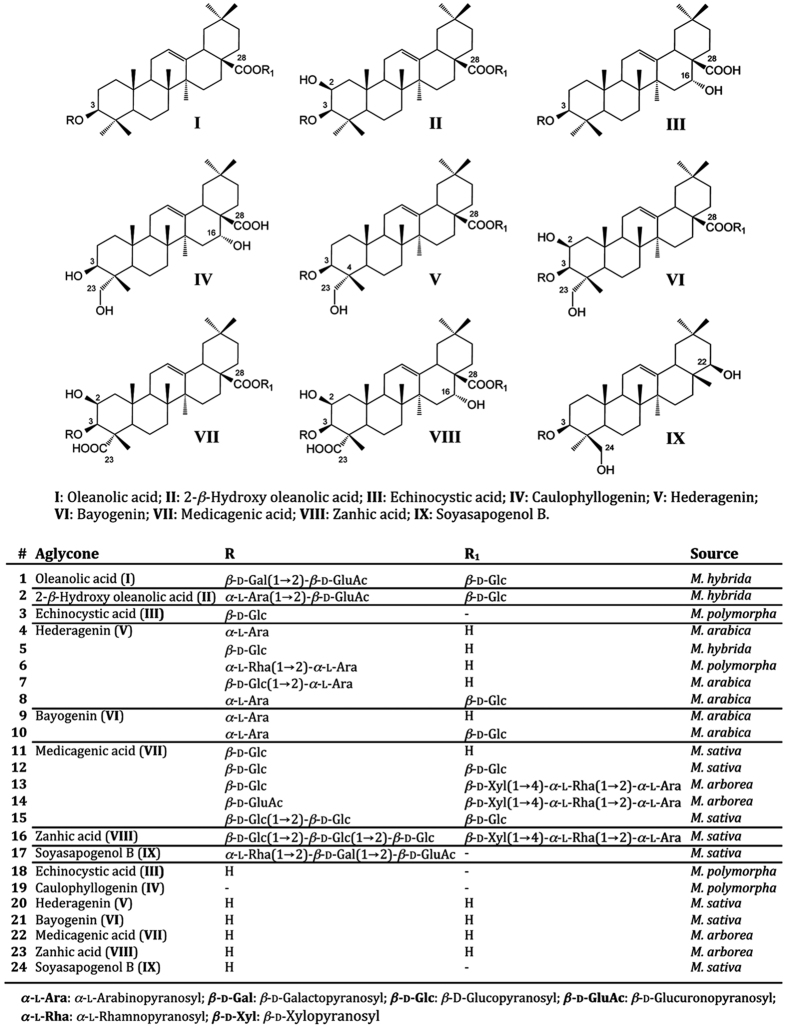
Structure and classification of saponins (**1–17**) and sapogenins (**18–24**) used in this investigation.

The affinity (K_d_) and rate constants (k_on_, k_off_) for PPARγ receptor/saponin or sapogenin interactions are reported in Table 1 and compared with the reference ligand LT175, whose K_d_ obtained by this analytical method (2.34 μM) basically confirmed that previously achieved from ITC experiments (3.66 μM)^24^. The kinetic analysis is reported in Supplementary Fig. 1. The compounds used in this study are reported in Fig. 1 and are both saponins (compounds **1–17**), and sapogenins (compounds **18–24**). For saponins, different values of affinity towards PPARγ were registered: higher affinity (K_d_ less than 100 μM) were found for saponins **1** (that shows the lowest equilibrium dissociation constant K_d_ = 18.33 μM), **3**, **4**, **6–8**, **10**, and **17**; a middle affinity (K_d_ 125–150 μM) was found for saponins **2**, **5** and **9**, while a very low or no affinity (K_d_ from about 500 to >1000 μM) was observed for saponins **11–16** (see Table 1). The first group of saponins, including compounds with high and middle PPARγ affinity, was composed by mono- and bidesmosides of oleanolic acid, 2*β*-hydroxy oleanolic acid, echinocystic acid, hederagenin, bayogenin and soyasapogenol B, whereas the second group of saponins showing low or no affinity with PPARγ, are all derivatives of medicagenic and zanhic acid.

Concerning sapogenins, only echinocystic acid (**18**) and caulophyllogenin (**19**) showed considerable affinities for PPARγ, with K_d_ = 9.70 and 54.82 μM, respectively, whereas the other tested sapogenins had very low or no affinity (K_d_ > 500, see Table 1).

**Figure 2:**
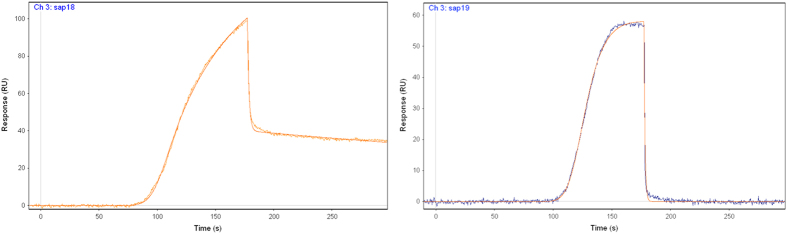
Representative data sets for kinetic analysis of sapogenin **18** and 19 in the interaction with PPARγ. Red lines represent the global fits of the data to a 1:1 bimolecular interaction model for **19** and a 2:1 model for **18**. The kinetic parameters obtained from each interaction are reported in Table 1.

Interestingly, echinocistic acid (**18**) showed a binding profile that differed from all the other tested compounds. In fact, the data obtained from kinetic analysis of **18** were more suitable for a 2:1 molecular interaction model (Fig. 2), suggesting that two molecules of **18** could simultaneously bind the receptor LBD. In addition, the high affinity binding site has a very small kinetic association rate constant k_on_ compared to that of the other compounds (144.8 M^−1^s^−1^ *versus* values ranging from 1·10^3^ to 7.6·10^4^ M^−1^s^−1^) and a lower dissociation rate constant k_off_ (1.4·10^−3^ *versus* 0.3–64 s^−1^), therefore a slow kinetics of association and dissociation can be hypothesized. A similar behavior has been observed for other nuclear receptor antagonists^25^.

By contrast, caulophyllogenin **19** showed a kinetic behavior characterized by a rapid association and dissociation time (Fig. 2).

### Transactivation activity

For saponins/sapogenins showing higher affinities, namely compounds **3**, **6**, **7**, **8**, **17**, **18** and **19**, transactivation activity at PPARγ has been evaluated on HepG2 cells. In this assay, only caulophyllogenin (**19**) and soyasaponin I (**17**) did not show cytotoxicity at concentrations up to 100 μM. The other tested compounds showed cytotoxic activities at values ranging from 3 to 20 μM (Table 2).

**Figure 3:**
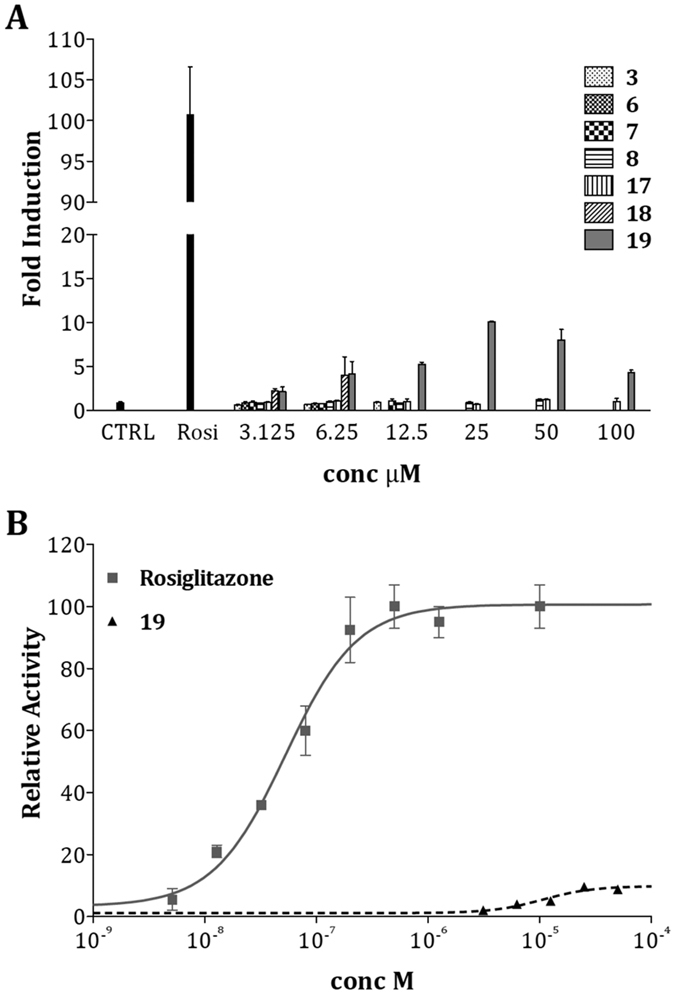
(**A**) PPARγ activity from saponins/sapogenins showing higher affinities (Rosi corresponds to the reference compound rosiglitazone); (**B**) Dose-response curve on PPARγ from rosiglitazone and sapogenin **19**.

With regard to PPARγ activity, we tested these compounds at doses lower than that at which they showed cytotoxicity (100 μM for soyasaponin I **17** and caulophyllogenin **19**, 50 μM for saponin **8**, 12.5 μM for saponins **3** and **7**, 6.25 μM for saponin **6** and echinocystic acid **18**). Only **19** behaved as a partial agonist with EC_50_ = 12.6 ± 2.7 μM and efficacy = 9.4 ± 0.6%. **18** displayed similar activity only at concentrations up to 6.25 μM (Fig. 3).

The antagonist behavior of the above compounds was also evaluated in displacement experiments against rosiglitazone. Only echinocystic acid **18** showed moderate activity reducing the effects of the reference compound by 40% at 5 μM.

### X-ray structure

**Figure 4:**
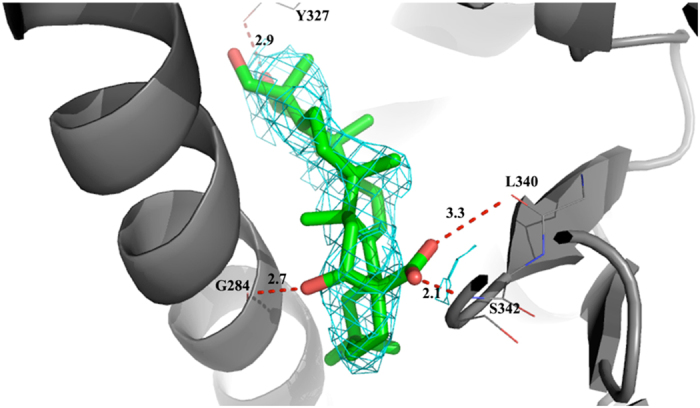
Binding of Sapogenin **19** in the LBD of PPARγ with 2Fo-Fc electron density map calculated around the ligand.

X-ray diffraction data were collected for the PPAR complexes with the two sapogenins **18** and **19** to provide an explanation at the molecular level for their different behavior as antagonist and partial agonist of PPARγ, respectively. Unfortunately, only the structure of the complex PPARγ/**19** was solved, whilst for PPARγ/**18** the electron density in the region of the ligand could not be clearly interpreted. The binding mode of **19** in the ligand binding domain (LBD) of PPARγ is shown in Fig. 4. The final omit map showed clear electron density in the LBD of PPARγ where one molecule of **19** can be easily fitted. The ligand was accommodated between the helix3 and the β-sheet, where its carboxylic oxygens formed a hydrogen bond (2.1 Å) with the NH group of S342, belonging to the β-sheet, and the CO of L340 (3.3 Å), respectively. The 16α-hydroxy group is H-bound to the CO of G284 on helix 3 (2.7 Å). The 3*β*-hydroxy group made a H-bond with the hydroxyl group of the Y327 side-chain (2.9 Å). Moreover, there were extensive vdW interactions between the sulphur atom of C285, on the helix 3, and the carbon atoms of the rings C and E of the ligand. The ring E was also engaged in vdW interactions with the aromatic ring of F363, on helix 7. The position of **19** in the LBD of PPARγ was very similar to that of the partial agonist *R* enantiomer of LT175 (pdb 3D6D^26^) (Supplementary Fig. 2a) and also to that of the fatty acid 13-(*S*)-HODE (pdb 2VST^27^), a natural agonist of PPARγ (Supplementary Fig. 2b).

### Isothermal titration calorimetry

ITC experiments were performed to confirm the interaction of **19** with PPARγ, comparing the thermodinamic parameters (ΔH, ΔS and K_d_) of this rigid sapogenin with those of the more flexible fatty acid 13-(*S*)-HODE. The results (Supplementary Fig. 3) indicated that **19** binds to the PPARγ-LBD with a K_d_ not far from that obtained by SPR techniques (6.5 vs 54.8 μM, respectively). Moreover, the comparison of the thermodynamic parameters between the two compounds showed that although the two compounds have similar K_d_ (6.5 and 9.4 μM for **19** and 13-(*S*)-HODE, respectively), the binding of 13-(*S*)-HODE, whose higher flexibility allows better interactions with the protein, shows a more favorable enthalpic contribution (ΔH = −2.21 versus −1.55 kcal/mol, respectively), instead the binding of **19** is associated to a more favorable entropic term (−TΔS = −5.46 versus −4.56 kcal/mol, respectively), also due to the minor loss of translational and rotational degrees of freedom of this more rigid ligand upon binding. Anyway, both binding interactions seem to be entropy-driven and more hydrophobic in character.

## Discussion

A selected series of previously purified saponins and sapogenins was screened by SPR techniques to test their affinity to PPARγ in order to select the most promising compounds for the activity test and for X-ray analysis. All the tested compounds had the same pentacyclic triterpene structure with differences in number and position of hydroxyl and carboxylic groups on the triterpene skeleton and nature and number of the sugars linked at C-3 and C-28 position.

The SPR experiment showed that, among saponins, the compounds with higher affinity were, in general, short sugar chains mono- and bidesmoside saponins characterized by the presence of a methyl or alcoholic group at C-23 position on the triterpenic nucleus. On the contrary, a carboxylic group in the same position, as in the derivatives of medicagenic and zanhic acid, significantly reduced the affinity. In addition, the hydroxyl group at the 2*β* position of the aglycone seemed to lower the affinity. Saponins of 2*β*-hydroxy oleanolic acid (compound **2**) and bayogenin (compounds **9** and **10**) showed, in general, higher dissociation constants K_d_ compared to saponins of oleanolic acid (compound **1**) and hederagenin (compounds **4**, **6** and **7**) in which the 2*β*-hydroxy group was not present.

The glycosidic portion of the molecule seemed to be an additional important feature for the interaction with PPARγ. As reported in Table 1, most saponins with higher affinity were glycosides of hederagenin (compounds **4**, **6**, **7** and **8**), bayogenin (compounds **9** and **10**) and soyasapogenol B (compound **17**) even though their aglycone portion, hederagenin (**20**), bayogenin (**21**) and soyasapogenol B (**24**), didn’t show affinity to the nuclear receptor. This allows to suggest that sugars, in particular when linked at the C-3 position, are of relevance for the interaction mechanisms with the binding site of PPARγ. Sugar chains could either allow a correct positioning of the aglycone moiety in the affinity site of the nuclear receptor or favor its accommodation into an alternative binding site. In fact, the X-ray structure of the complex PPARγ/**19** showed that there could be additional space for the accommodation of the glycoside moieties in the region tipically occupied by full agonists, towards the helix 12.

Also the sugar chain length, in particular at C-28 position, seems to be of importance to improve affinity. The most active saponins are both monodesmosidic or monosaccaride C-28 substituted compounds.

Concerning sapogenins, the SPR experiment showed that only echinocystic acid (**18**) and caulophyllogenin (**19**) possessed a good affinity for the PPARγ LBD (for **19** this was also confirmed by ITC analysis). Both compounds are characterized by the presence of a 16*α*-hydroxyl group in the molecule that probably is another important feature in conferring affinity to PPARγ. The same 16α-hydroxy substituent is also present in zanhic acid but this character is probably not sufficient to contrast the strong binding inhibition performed by the carboxylic group at C-23 position.

In conclusion, some chemical characteristics of substrates were of relevance in conferring binding affinity to PPARγ. The presence of a methyl or hydroxyl at C-23 position of the triterpenic nucleus strongly increased affinity, which was inhibited from the presence of a carboxylic group at the same position. The 2*β*-hydroxy substitution seemed to lower the affinity, while the 16α-hydroxy substitution had some positive effects on affinity only if no carboxylic group was present at C-23 position. Moreover, the glycosylation at C-3 seemed to increase the activity, although it should be taken into account the possibility that oral administrations of saponins might lead to hydrolysis of glycosides from terpenoid.

The MTT assay test and the transcriptional activity test performed on the most affine compounds (saponins **3**, **6**, **7**, **8**, **17,** echinocystic acid **18** and caulophyllogenin **19**) indicated that **17** and **19** did not show any cytotoxic activity at concentrations up to 100 μM concentration, but only caulophyllogenin **19** showed a partial agonist activity towards PPARγ. Its low potency and efficacy (EC_50_ = 12.6 ± 2.7 μM, efficacy = 9.4 ± 0.6%) should not be underestimated because very similar to those of the well-known selective modulator metaglidasen^28^,^29^, a compound investigated as a useful agent for the treatment of type 2 diabetes and hyperglycemia, showing reduced adverse side effects. The X-ray structure of the complex PPARγ/**19** showed an accommodation of the ligand in the LBD between helix3 and the β-sheet, typical of known partial agonists, where it formed H-bonds with the first strand of the β-sheet and with helix3. Hypothetically, in this orientation of the ligand it could be very easy to accommodate glycoside moieties linked to the carbon atom 3 that protrude towards the helix12, in the region occupied by full agonists.

Very interestingly, echinocystic acid **18** showed antagonist activity towards PPARγ, a property that could be useful to inhibit the adipocyte differentiation which is a typical adverse effect of PPARγ agonists. The cytotoxic activity *in vitro* of **18** against human cancer cell lines (HepG2) deserves to be deepened to establish a possible role of this sapogenin as potential anti-cancer in combination with other anti-tumorigenic drugs, as already evidenced in previous papers^30^,^31^.

Echinocystic acid **18** and caulophyllogenin **19** used in this investigation were extracted from *M. polimorpha* (*Leguminose* family), but they are also components of *Crysantellum americanum*, a plant of the *Asteraceae* family which contains several triterpen saponins, whose therapeutic properties as hypolipidemic and hepatoprotective agents are known^32^. Further studies should be also performed to evaluate the anti-inflammatory activity of these sapogenins as possible inhibitors of NF-kB.

In conclusion, the present study confirm the interest on saponins and sapogenins as a valuable natural resource exploitable in the medical and food industry for ameliorating type 2 diabetes, obesity, metabolic syndrome and inflammation^33^. Particularly, we focused the interest on caulophyllogenin **19** as a promising partial agonist of PPARγ and echinocistic acid **18** as PPARγ antagonist.

Finally, the SPR analysis has been proved to be a very powerful and fast method for screening a large number of compounds and selecting the better candidates as agonists and also antagonists of the nuclear receptor PPARγ.

## Methods

### Extraction, purification and characterization of saponins and sapogenins from *Medicago* species

Saponins were extracted and purified as previously described^34^,^35^,^36^,^37^,^38^. Pure saponins were obtained by a combination of chromatographic separation steps (silica gel open column chromatography and semi-preparative Reversed Phase-High Performance Liquid Chromatography, RP-HPLC) from the crude saponin mixture from leaves and roots of *Medicago* species as described by Tava *et al*.^35^,^36^,^37^. Acid hydrolysis of saponin mixture afforded the related sapogenins that were obtained in a pure form after silica gel open column chromatography^23^. Detailed structural elucidation of pure saponins was obtained by Nuclear Magnetic Resonance (NMR) and Electrospray Ionization/Mass Spectrometry (ESI-MS/MS). Sapogenins were also evaluated by Gas Chromatography/Mass Spectrometry (GC/MS) analysis as their methyl-trimethylsilyl derivatives^34^,^35^,^36^,^37^,^39^. Saponins and sapogenins were dissolved in DMSO and used for the tests at the indicated concentrations.

### Protein preparation and crystallization

PPARγ LBD was expressed as N-terminal His-tagged proteins using a pET28 vector and then purified as previously described^40^. Briefly, freshly transformed *E. coli* BL21 DE3 were grown in LB medium with 30 μg of kanamycin/mL at 310 K to an OD of 0.6. The culture was then induced with 0.1 mM isopropyl-β-D-thio-galactopyranoside and further incubated at 291 K for 20 h. Cells were harvested and resuspended in a 20 mL/liter culture of Buffer A (20 mM Tris, 150 mM NaCl, 10% glycerol, 1 mM Tris 2-carboxyethylphosphine HCl (TCEP), pH 8) in the presence of protease inhibitors (Complete Mini EDTA-free; Roche Applied Science). Cells were sonicated, and the soluble fraction was isolated by centrifugation (35,000 × g for 45 min). The supernatant was loaded onto a Ni^2+^ -nitrilotriacetic acid column (GE Healthcare) and eluted with a gradient of imidazole 0–300 mM in Buffer A (batch method). The pure protein was identified by SDS PAGE. The protein was then dialyzed over buffer A to remove imidazole, and it was cleaved with thrombin protease (GE Healthcare) (10 units/mg) at room temperature for 2 h. The digested mixture was reloaded onto a Ni^2+^ -nitriloacetic acid column to remove His tag and the undigested protein. The flow-through was loaded onto a Q-Sepharose HP column (GE Healthcare) and eluted with a gradient of NaCl 0–500 mM in Buffer B (20 mM Tris, 10% glycerol, 1 mM TCEP, pH 8) with a BioLogic DuoFlow FPLC system (Bio-Rad Laboratories, Italy). Finally, the protein was purified by gel-filtration chromatography on a HiLoad Superdex 75 column (GE Healthcare) and eluted with Buffer C (20 mM Tris, 1 mM TCEP, 0.5 mM EDTA, pH 8). The protein was then concentrated at 8 mg/mL using Amicon centrifugal concentrators with a 10 kDa cutoff membrane (Millipore, USA). Part of the protein was used for SPR experiments. Crystals of apo-PPARγ were obtained by vapor diffusion at 18 °C using a sitting drop made by mixing 2 μL of protein solution with 2 μL of reservoir solution (0.8 M Na Citrate, 0.15 M Tris, pH 8.0). The crystals were soaked for 8 days in a storage solution (1.2 M Na Citrate, 0.15 M Tris, pH 8.0) containing the sapogenin **19** (0.25 mM). The ligand dissolved in DMSO was diluted in the storage solution so that the final concentration of DMSO was 0.5%. The storage solution with glycerol 20% (v/v) was used as cryoprotectant. Crystals of PPARγ/**19** belong to the space group *C2* with cell parameters shown in Table 3.

### Data collection, structure determination and refinement

X-ray data set were collected at 100 K under a nitrogen stream using sinchrotron radiation (beamline ID 23-2 at ESRF, Grenoble, France). The collected data were processed using the programs MOSFLM and SCALA^41^. Structure solution was performed with AMoRe^42^, using the coordinates of PPARγ/LT175 (PDB code 3B3K) as the starting model^27^. The coordinates were then refined with CNS^43^. All data between 50.00 and 2.25 Å were included for PPARγ/**19** belonging to *C2* space group. A final step of refinement was performed with the software Phenix^44^. The statistics of crystallographic data and refinement are summarized in Table 3. The coordinates of PPARγ/**19** have been deposited in the Brookhaven Protein Data Bank (PDB) with the code 5F9B.

### Surface Plasmon Resonance

Surface plasmon resonance analyses were performed by using Pioneer AE optical biosensor equipped with COOH5 chips (SensiQ). PPARγ surfaces were prepared by using standard amine-coupling procedures^45^ and HBS (Hepes-buffered saline: 20 mM Hepes, 150 mM sodium chloride, 0.005% P20, pH 7.4) as the running buffer. Flow cells were activated for 7 min by injecting 140 μL of 50 mM *N*-hydroxysuccinimide (NHS):200 mM ethyl-3(3-dimethylamino) propyl carbodiimide (EDC). Fifty μL of a 0.25 mg/mL PPARγ solution (in 10 mM sodium acetate, pH 5.0) were injected for 5 min at 10 μL/min on channels 1 and 3 (channel 2 was used as ref. 3, for a duplicate experiment), followed by a 70-μL injection of ethanolamine to block any remaining activated groups on the surface. More then 13,500 RU of protein were immobilized on both channels. The stability of the PPARγ surface was demonstrated by the flat baseline achieved at the beginning (0–60 s) of each sensorgram. The screening of the analytes (saponins and sapogenins) was performed using HBS without P20, with 1 mM DTT and 2% DMSO. To collect detailed kinetic data the OneStep^46^,^47^,^48^ protocol was used, injecting the analytes at a flow rate of 50 μL/min and at the concentration of 100 μM (50 μM for **14** and **15**) over the three channels at 20 °C (association phase of 180 s). Five buffer blanks were injected for double referencing . The regeneration of the surfaces between binding cycles was not necessary because all the analytes dissociate quickly in the 120 s dissociation phase. A DMSO calibration plot was constructed (buffer sample containing 1–3% (vol/vol) DMSO) to correct for bulk refractive index shifts^49^. Data were collected at a rate of 20 Hz. All sensorgrams were processed by using double referencing^50^. First, the responses from the reference surface (channel 2) were subtracted from the binding responses collected over the reaction surfaces to correct for bulk refractive index changes. Second, the response from an average of the blanks was subtracted to remove any systematic artifact observed between the reaction and the reference flow cells. To obtain kinetic rate constants and affinity constants the corrected response data were fit in the program QDAT. A kinetic analysis of each ligand/analyte interaction was obtained by fitting the response data to a reversible 1:1 bimolecular interaction model (but for few analytes a 2:1 interaction model was used). The equilibrium dissociation constant (K_d_) was determined by the ratio k_off_/k_on_. Constant reported in Table 1 represent the average of two independent analyses of each PPARγ/saponine interaction.

### Isothermal titration calorimetry

ITC experiments were performed at 22 °C by using a ITC200 microcalorimeter (MicroCal, Inc., Northampton, MA, USA). PPARγ was extensively dialyzed against a solution of HEPES (20 mM, pH 8.0) and TCEP (1 mM) with Amicon Ultra filters, and the final exchange buffer was used to dilute 13-(*S*)-HODE (10 μg with 83 μL of buffer to obtain a 400 μM solution) and the SAP19 stock solution (25 mM in DMSO). DMSO was added to the protein solution at the same percentage of the ligand solution (2%) for the experiment with SAP19. The protein solution (50 μM) was placed in the sample cell, and ligand solution (500 μM) was loaded into the syringe injector (PPARγ 40 μM and the ligand 400 μM for the experiment with 13-HODE) . The titrations involved 19 injections of 2 μL each at 150 s intervals. A reference titration of ligand into buffer was used to correct for heats of dilution. Thermodynamic data was processed with Origin 7.0 software (MicroCal). Fitting the isotherms with one-site binding model yealded the values of the association constant (K_a_).

### Transactivation assay

#### Plasmids

The expression vectors expressing the chimeric receptor containing the yeast Gal4-DNA binding domain fused to the human PPARα- or PPARγ-LBD and the reporter plasmid for these Gal4 chimeric receptors (pGal5TKpGL3) containing five repeats of the Gal4 response elements upstream of a minimal thymidine kinase promoter that is adjacent to the luciferase gene were described previously^51^.

#### Cell culture, plasmid

Human hepatocellular liver carcinoma cell line HepG2 (Interlab Cell Line Collection, Genoa, Italy) was cultured in Minimum Essential Medium (MEM) containing 10% heat-inactivated fetal bovine serum, 100 U·mL^−1^ of penicillin G, and 100 μg·mL^−1^ of streptomycin sulfate at 37 °C in a humidified atmosphere of 5% CO_2_.

### Transfections and luciferase-based transactivation assays

For transactivation assays, 1·10^5^ cells per well were seeded in a 96-well plate and transfected after 24 h with K2 Transfection System (Biontex Laboratories GmbH), according to the manufacturer’s protocol using 0.20 μg/well of DNA. Cells were transfected with expression plasmids encoding the fusion protein Gal4-PPARα-LBD or Gal4-PPARγ-LBD, pGal5TKpGL3, and pCMVβgal to normalize the transfection efficacy. 24 h after transfection, medium was replaced with fresh complete growth medium supplemented with test compounds (ranging from 200 nM to 100 μM), reference compounds Wy-14,643 (10 μM) and rosiglitazone (2 μM), or DMSO 0.1%. After further 24 h of incubation cells were lysed and luciferase activity in cell extracts was determined by a luminometer (VICTOR^3^ V Multilabel Plate Reader, PerkinElmer, Monza, Italy) and normalized for β-galactosidase activity. Fold induction activity was calculated and plotted using GraphPad Prism5 software.

### MTT assay for cell viability

Cell viability was measured using the MTT assay. HepG2 cells were seeded at a density of 1·10^5^ cells/well into 96-well flat bottom culture plates containing test compounds (ranging from 200 nM to 100 μM final concentration), in a final volume of 100 μL. Test compounds were dissolved in DMSO (<0.4% final concentration; DMSO carrier had no effect on cell proliferation). Control wells lacked inhibitor. After 24 h of incubation at 37 °C in a 5% CO_2_ atmosphere, 3-(4,5-dimethylthiazole-2-yl)-2,5-diphenyltetrazolium bromide (MTT, 5 mg/mL stock solution) was added to a final concentration of 0.5 mg/mL. To control for background absorbance, six wells of cells were lysed by adding Triton X-100 (0.1% v/v final concentration) immediately prior to the addition of MTT reagent. After incubation under the same conditions for further 3–4 h, the culture medium was removed, the insoluble product dissolved by the addition of 100 μL of solvent (50% DMSO, 50% EtOH v/v), and the absorbance of the well was measured at 570 nm using a PERKIN–ELMER Victor V^3^ plate reader. Cell growth inhibition was then calculated using the following Equation,


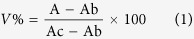


where *V%* is the percentage of cell viability, *A* is the absorbance of treated cultures, *Ab* is the absorbance of background control, and *Ac* is the absorbance of control cultures.

### Statistics

All experiments were performed in triplicate and were repeated at least twice with similar results. The results were expressed as mean ± SEM. The responses to agonists were calculated as a percentage compared to control, which was set to 100%.

## Additional Information

**Accession codes:** The coordinates of PPARγ /**19** have been deposited in the Brookhaven Protein Data Bank (PDB) with the code 5F9B.

**How to cite this article**: Montanari, R. *et al*. Screening of saponins and sapogenins from *Medicago* species as potential PPARγ agonists and X-ray structure of the complex PPARγ /caulophyllogenin. *Sci. Rep*. **6**, 27658; doi: 10.1038/srep27658 (2016).

## Supplementary Material

Supplementary Information

## Figures and Tables

**Table 1:** Affinity (K_d_) and rate constants (k_on_, k_off_) for PPARγ receptor/saponin (or sapogenin) interactions.

Interaction	K_on_ (M^−1^s^−1^)^a^	K_off_(s^−1^)^a^	K_d_(μM)
LT175b,^c^	3.5(0.1)e5 9.5(0.2)e2	0.82(0.04) 0.81(0.02)	2.34 (0.04) 850 (30)
SAP1	6.12(0.04)e4	1.122(0.002)	18.33(0.09)
SAP2	1.254(0.003)e4	1.572(0.004)	125.34(0.04)
SAP3	1.567(0.003)e4	1.264(0.003)	80.67(0.03)
SAP4	1.333(0.003)e4	0.694(0.002)	52.05(0.05)
SAP5	1.040(0.002)e3	1.381(0.003)	132.71(0.05)
SAP6	1.346(0.004)e4	0.913(0.002)	67.85(0.06)
SAP7	2.928(0.007)e4	1.260(0.003)	43.03(0.03)
SAP8	3.74(0.01)e4	2.139(0.008)	57.18(0.04)
SAP9	5.18(0.02)e3	0.769(0.002)	148.25(0.09)
SAP10	1.626(0.004)e4	1.451(0.004)	89.28(0.04)
SAP11	1.949(0.006)e3	0.970(0.003)	497.4(0.2)
SAP12^d^	–	–	–
SAP13	5.64(0.03)e3	4.13(0.03)	731.2(0.2)
SAP14	–	–	>1000
SAP15	–	–	>1000
SAP16	–	–	>1000
SAP17^c^	5.1(0.1)e4	1.68(0.04)	32.5(0.02)
SAP18^c^	144.8(0.2) 1.98(0.01)e4	1.405(0.004)e-3 0.715(0.002)	9.70(0.03) 36.1(0.2)
SAP19	2.053(0.005)e4	1.126(0.002)	54.82(0.03)
SAP20	–	–	>1000
SAP21^d^	–	–	–
SAP22	4.65(0.04)e3	2.47(0.02)	531(7)
SAP23	4.76(0.08)e3	3.33(0.05)	699.2(0.6)
SAP24^d^	–	–	–

^a^Experimental error is reported in parentheses.

^b^LT175 was chosen as reference compound because its K_d_ is known from ITC experiments (K_d_ = 3.66 μM).

^c^For this compound data were fit to a 2:1 molecular interaction model.

^d^For this compound data didn’t fit to models.

**Table 2:** Cytoxicity values (MTT assay, see Methods) on HepG2 from saponins/sapogenins showing higher affinities.

Compd	IC_50_ [μM]
**3**	7.8 ± 0.3
**6**	3.5 ± 0.1
**7**	12.3 ± 0.2
**8**	20.2 ± 1.8
**17**	>100
**18**	4.1 ± 0.4
**19**	>100

IC_50_ represents the concentration that reduces the cell viability by 50%.

**Table 3:** Statistics of Crystallographic Data and Refinement.

	PPARγ/SAP19
Space group	*C2*
Wavelenght (Å)	0.8726
Temperature (K)	100
Cell axes (Å)	93.21; 61.66; 118.9
Beta angle (°)	102.8
Resolution range (Å)	50.00–2.25 (2.30–2.25)
Rmerge (%)	6.0 (34.9)
Unique reflections	31203
I/σ(I)	9.2 (2.8)
Multiplicity	3.6 (3.4)
Completeness (%)	99.3 (99.5)
R_factor_ (%)	23.2
R_free_ (%)	28.1

*The values in parentheses refer to the outer shell.

## References

[b1] Berger J. P., Akiyama T. E. & Meinke P. T. PPARs: therapeutic targets for metabolic disease. Trends Pharmacol Sci 26, 244–251, doi: 10.1016/j.tips.2005.03.003 (2005). 15860371

[b2] Berger J. & Moller D. E. The mechanisms of action of PPARs. Annu Rev Med 53, 409–435, doi: 10.1146/annurev.med.53.082901.104018 (2002). 11818483

[b3] Kliewer S. A. . Fatty acids and eicosanoids regulate gene expression through direct interactions with peroxisome proliferator-activated receptors alpha and gamma. Proc Natl Acad Sci USA 94, 4318–4323, doi: 10.1073/pnas.94.9.4318 (1997). 9113987 PMC20720

[b4] Oakes N. D. . A new antidiabetic agent, BRL 49653, reduces lipid availability and improves insulin action and glucoregulation in the rat. Diabetes 43, 1203–1210, doi: 102337/diab.43.10.1203 (1994). 7926289 10.2337/diab.43.10.1203

[b5] Nesto R. W. . Thiazolidinedione use, fluid retention, and congestive heart failure: a consensus statement from the American Heart Association and American Diabetes Association. Diabetes Care 27, 256–263, doi: 10.2337/diacare.27.1.256 (2004). 14693998

[b6] Kahn S. E. . Rosiglitazone-associated fractures in type 2 diabetes: an Analysis from A Diabetes Outcome Progression Trial (ADOPT). Diabetes Care 31, 845–851, doi: 10.2337/dc07-2270 (2008). 18223031

[b7] Wright H. M. . A synthetic antagonist for the peroxisome proliferator-activated receptor gamma inhibits adipocyte differentiation. J Biol Chem 275, 1873–1877, doi: 10.1074/jbc.275.3.1973 (2000). 10636887

[b8] Waki H., Yamauchi T. & Kadowaki T. PPARgamma antagonist as a potential drug for the treatment of obesity and diabetes. Nihon Rinsho 68, 350–355 (2010). 20158108

[b9] Hotamisligil G. S. Inflammation and metabolic disorders. Nature 444, 860–867, doi: 10.1038/nature05485 (2006). 17167474

[b10] Lee K. T. . Hypoglycemic and hypolipidemic effects of tectorigenin and kaikasaponin III in the streptozotocin-lnduced diabetic rat and their antioxidant activity *in vitro*. Arch Pharm Res 23, 461–466, doi: 10.1007/bf02976573 (2000). 11059824

[b11] Liu Y. W. . Ginsenoside Re attenuates diabetes-associated cognitive deficits in rats. Pharmacol Biochem Behav 101, 93–98, doi: 10.1016/j.pbb.2011.12.003 (2012). 22197711

[b12] Bhavsar S. K., Singh S., Giri S., Jain M. R. & Santani D. D. Effect of saponins from Helicteres isora on lipid and glucose metabolism regulating genes expression. J Ethnopharmacol 124, 426–433, doi: 10.1016/j.jep.2009.05.041 (2009). 19505560

[b13] Eu C. H., Lim W. Y., Ton S. H. & bin Abdul Kadir K. Glycyrrhizic acid improved lipoprotein lipase expression, insulin sensitivity, serum lipid and lipid deposition in high-fat diet-induced obese rats. Lipids Health Dis 9, 81, doi: 10.1186/1476-511X-9-81 (2010). 20670429 PMC2927592

[b14] Hu X. . Dietary saponins of sea cucumber ameliorate obesity, hepatic steatosis, and glucose intolerance in high-fat diet-fed mice. J Med Food 15, 909–916, doi: 10.1089/jmf.2011.2042 (2012). 22897583

[b15] Lee K. T. . The antidiabetic effect of ginsenoside Rb2 via activation of AMPK. Arch Pharm Res 34, 1201–1208, doi: 10.1007/s12272-011-0719-6 (2011). 21811928

[b16] Hostettmann K. M. A. *Chemistry and pharmacology of natural products: Saponins*. Cambridge University Press, UK edn (1995).

[b17] Kwon D. Y. . Platyconic acid, a saponin from Platycodi radix, improves glucose homeostasis by enhancing insulin sensitivity *in vitro* and *in vivo*. Eur J Nutr 51, 529–540, doi: 10.1007/s00394-011-0236-x (2012). 21847688

[b18] Lee E. J., Kang M. & Kim Y. S. Platycodin D inhibits lipogenesis through AMPKalpha-PPARgamma2 in 3T3-L1 cells and modulates fat accumulation in obese mice. Planta Med 78, 1536–1542, doi: 10.1055/s-0032-1315147 (2012). 22872592

[b19] Li W. . Anti-Inflammatory and PPAR Transactivational Effects of Oleanane-Type Triterpenoid Saponins from the Roots of Pulsatilla koreana. Biomol Ther (Seoul) 22, 334–340, doi: 10.4062/biomolther.2014.047 (2014). 25143813 PMC4131524

[b20] Zhang Y. . Protopanaxatriol, a novel PPARgamma antagonist from Panax ginseng, alleviates steatosis in mice. Sci Rep 4, 7375, doi: 10.1038/srep07375 (2014). 25487878 PMC4260220

[b21] Wong H. R. & Menendez I. Y. Sesquiterpene lactones inhibit inducible nitric oxide synthase gene expression in cultured rat aortic smooth muscle cells. Biochemical and biophysical research communications 262, 375–380, doi: 10.1006/bbrc.1999.1207 (1999). 10462483

[b22] Ley K., Laudanna C., Cybulsky M. I. & Nourshargh S. Getting to the site of inflammation: the leukocyte adhesion cascade updated. Nat Rev Immunol 7, 678–689, doi: 10.1038/nri2156 (2007). 17717539

[b23] Avato P. . Antimicrobial activity of saponins from *Medicago* sp.: structure-activity relationship. Phytotherapy research: PTR 20, 454–457, doi: 10.1002/ptr.1876 (2006). 16619355

[b24] Calleri E. . Frontal affinity chromatography with MS detection of the ligand binding domain of PPARgamma receptor: ligand affinity screening and stereoselective ligand-macromolecule interaction. J Chromatogr A 1232, 84–92, doi: 10.1016/j.chroma.2011.10.037 (2012). 22056242

[b25] Rich R. L. . Kinetic analysis of estrogen receptor/ligand interactions. Proceedings of the National Academy of Sciences of the United States of America 99, 8562–8567, doi: 10.1073/pnas.142288199 (2002). 12077320 PMC124311

[b26] Montanari R. . Crystal structure of the peroxisome proliferator-activated receptor gamma (PPARgamma) ligand binding domain complexed with a novel partial agonist: a new region of the hydrophobic pocket could be exploited for drug design. J Med Chem 51, 7768–7776, doi: 10.1021/jm800733h (2008). 19053776

[b27] Itoh T. . Structural basis for the activation of PPARg by oxidized fatty acids. Nat Struct Mol Biol 15, 924–31, doi: 10.1038/nsmb.1474 (2008). 19172745 PMC2939985

[b28] Aronow W. S. . Effect of halofenate on serum lipids. Clin Pharmacol Ther 14, 358–365 (1973). 4572798 10.1002/cpt1973143358

[b29] Laghezza A. . On the metabolically active form of metaglidasen: improved synthesis and investigation of its peculiar activity on peroxisome proliferator-activated receptors and skeletal muscles. ChemMedChem 10, 555–565, doi: 10.1002/cmdc.201402462 (2015). 25641779

[b30] Podolak I., Galanty A. & Sobolewska D. Saponins as cytotoxic agents: a review. Phytochem Rev 9, 425–474, doi: 10.1007/s11101-010-9183-z (2010). 20835386 PMC2928447

[b31] Tong X., Lin S., Fujii M. & Hou D. X. Molecular mechanisms of echinocystic acid-induced apoptosis in HepG2 cells. Biochemical and biophysical research communications 321, 539–546, doi: 10.1016/j.bbrc.2004.07.004 (2004). 15358141

[b32] Honore-Thorez D. Description, identification and therapeutic use of Chrysanthellum “americanum”: Chrysanthellum indicum DC. subsp afroamericanum B. L. Turner. J Pharm Belg 40, 323–331 (1985). 4087125

[b33] Ming L. J. & Yin A. C. Therapeutic effects of glycyrrhizic acid. Nat Prod Commun 8, 415–418 (2013). 23678825

[b34] Bialy Z., Jurzysta M., Oleszek W., Piacente S. & Pizza C. Saponins in alfalfa (*Medicago* sativa L.) root and their structural elucidation. Journal of agricultural and food chemistry 47, 3185–3192 (1999). 10552628 10.1021/jf9901237

[b35] Tava A. . Triterpenoid glycosides from leaves of *Medicago* arborea L. Journal of agricultural and food chemistry 53, 9954–9965, doi: 10.1021/jf052468x (2005). 16366680

[b36] Tava A. . New triterpenic saponins from the aerial parts of *Medicago* arabica (L.) huds. Journal of agricultural and food chemistry 57, 2826–2835, doi: 10.1021/jf8036984 (2009). 19256537

[b37] Tava A., Pecetti L., Romani M., Mella M. & Avato P. Triterpenoid glycosides from the leaves of two cultivars of *Medicago* polymorpha L. Journal of agricultural and food chemistry 59, 6142–6149, doi: 10.1021/jf2005854 (2011). 21526796

[b38] Bialy Z., Jurzysta M., Mella M. & Tava A. Triterpene saponins from the roots of *Medicago* hybrida. Journal of agricultural and food chemistry 54, 2520–2526, doi: 10.1021/jf0581628 (2006). 16569038

[b39] Tava A. & Pecetti L. Chemical investigation of saponins from twelve annual *Medicago* species and their bioassay with the brine shrimp Artemia salina. Nat Prod Commun 7, 837–840 (2012). 22908560

[b40] Pochetti G. . Insights into the mechanism of partial agonism: crystal structures of the peroxisome proliferator-activated receptor gamma ligand-binding domain in the complex with two enantiomeric ligands. J Biol Chem 282, 17314–17324, doi: 10.1074/jbc.M702316200 (2007). 17403688

[b41] Battye T. G., Kontogiannis L., Johnson O., Powell H. R. & Leslie A. G. iMOSFLM: a new graphical interface for diffraction-image processing with MOSFLM. Acta Crystallogr D Biol Crystallogr 67, 271–281, doi: 10.1107/S0907444910048675 (2011). 21460445 PMC3069742

[b42] Navaza J. AMoRe: an automated package for molecular replacement. Acta Crystallogr A50, 157–163 (1994).

[b43] Brunger A. T. . Crystallography & NMR system: A new software suite for macromolecular structure determination. Acta Crystallogr D Biol Crystallogr 54, 905–921, doi: 10.1107/s0907444998003254 (1998). 9757107

[b44] Adams P. D. . PHENIX: a comprehensive Python-based system for macromolecular structure solution. Acta Crystallogr D Biol Crystallogr 66, 213–221, doi: 10.1107/S0907444909052925 (2010). 20124702 PMC2815670

[b45] Jonsson U. & Malmqvist M. Real time biospecific interaction analysis. Adv Biosens 2, 291–336, (1992).

[b46] Quinn J. G. Modeling Taylor dispersion injections: determination of kinetic/affinity interaction constants and diffusion coefficients in label-free biosensing. Anal Biochem 421, 391–400, doi: 10.1016/j.ab.2011.11.024 (2012). 22197421

[b47] Quinn J. G. Evaluation of Taylor dispersion injections: determining kinetic/affinity interaction constants and diffusion coefficients in label-free biosensing. Anal Biochem 421, 401–410, doi: 10.1016/j.ab.2011.11.023 (2012). 22197422

[b48] Rich R. L., Quinn J. G., Morton T., Stepp J. D. & Myszka D. G. Biosensor-based fragment screening using FastStep injections. Anal Biochem 407, 270–277, doi: 10.1016/j.ab.2010.08.024 (2010). 20800052 PMC2949542

[b49] Frostell-Karlsson A. . Biosensor analysis of the interaction between immobilized human serum albumin and drug compounds for prediction of human serum albumin binding levels. J Med Chem 43, 1986–1992, doi: 10.1021/jm991174y (2000). 10821711

[b50] Myszka D. G. M. T. A. CLAMP: a biosensor kinetic data analysis program. Trends Biochem. Sci. 23, 149–150 (1998). 9584619 10.1016/s0968-0004(98)01183-9

[b51] Raspe E. . Modulation of rat liver apolipoprotein gene expression and serum lipid levels by tetradecylthioacetic acid (TTA) via PPARalpha activation. J Lipid Res 40, 2099–2110 (1999). 10553013

