# Supplementary material for: Screening of saponins and sapogenins from Medicago species as potential PPARγ agonists and X-ray structure of the complex PPARγ/caulophyllogenin
Source: Sci Rep. 2016 Jun 10;6:27658. doi: 10.1038/srep27658 (PMC4901321; doi:10.1038/srep27658)
Supplement: Supplementary Information [file srep27658-s1.pdf]

Screening of saponins and sapogenins from *Medicago* species as potential PPARγ agonists and X-ray structure of the complex PPARγ/caulophyllogenin

Roberta Montanari, Davide Capelli, Aldo Tava, Andrea Galli, Antonio Laghezza, Paolo Tortorella, Fulvio Loiodice & Giorgio Pochetti*

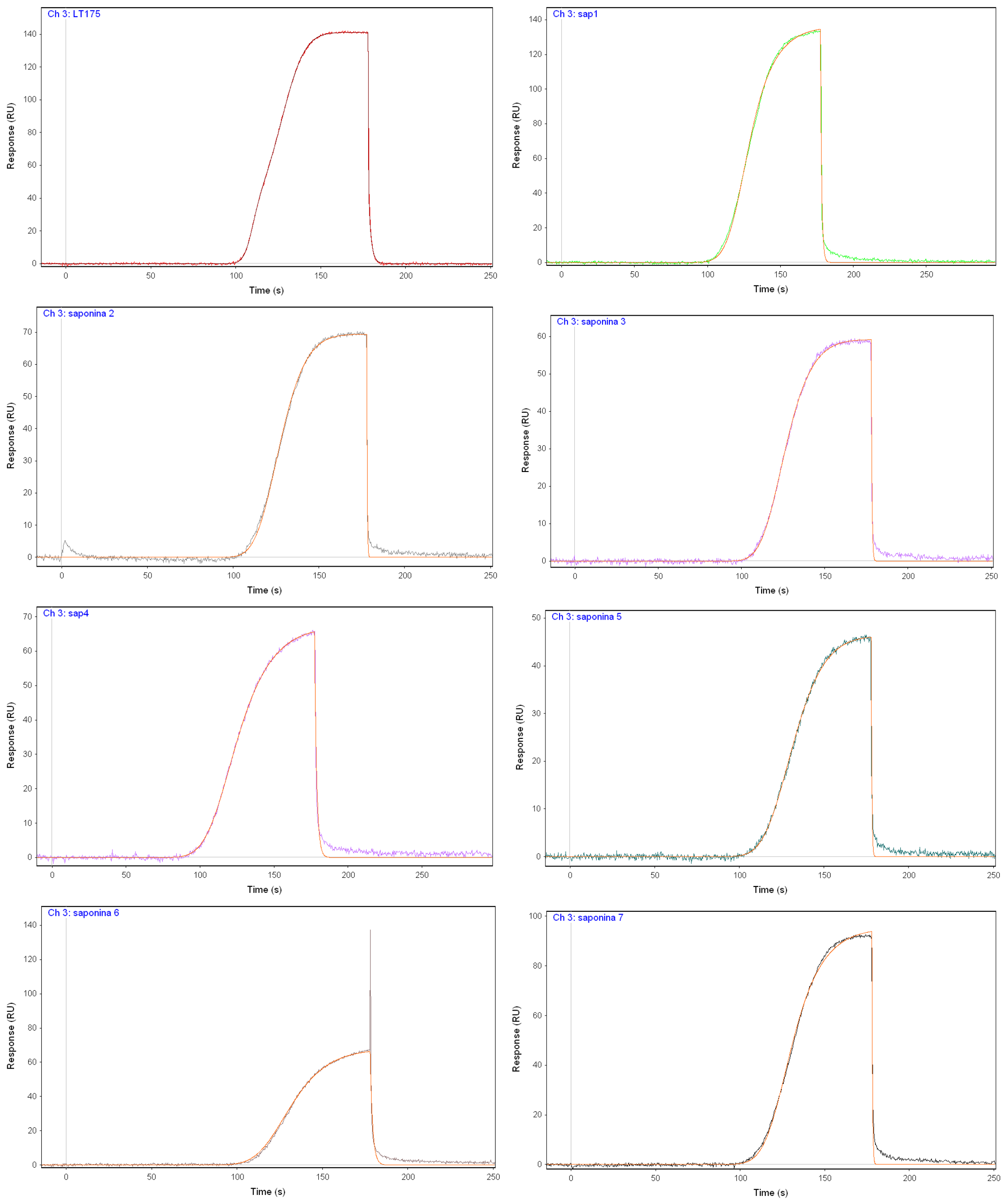

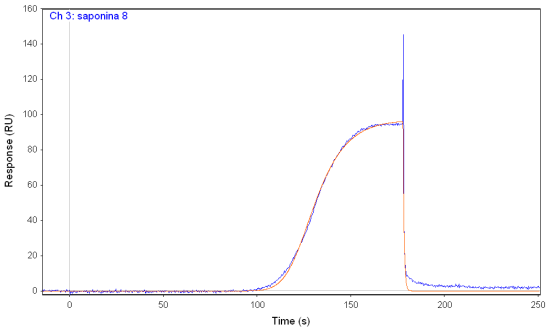
Ch 3: saponina 8
Response (RU)
Time (s)

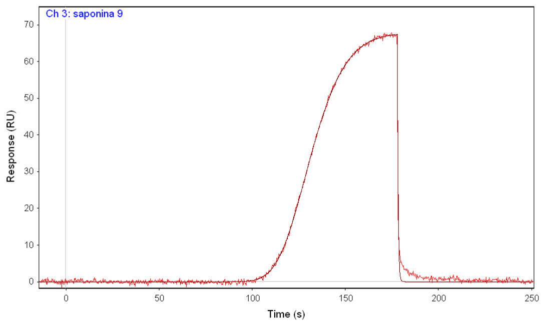
Ch 3: saponina 9
Response (RU)
Time (s)

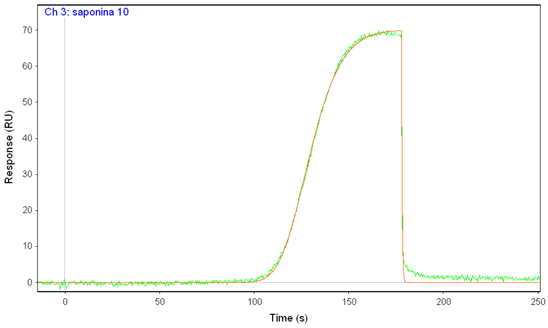
Ch 3: saponina 10
Response (RU)
Time (s)

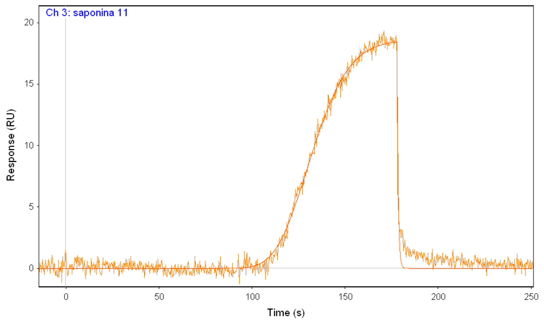
Ch 3: saponina 11
Response (RU)
Time (s)

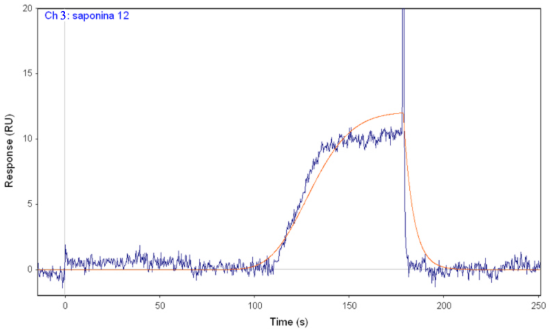
Ch 3: saponina 12
Response (RU)
Time (s)

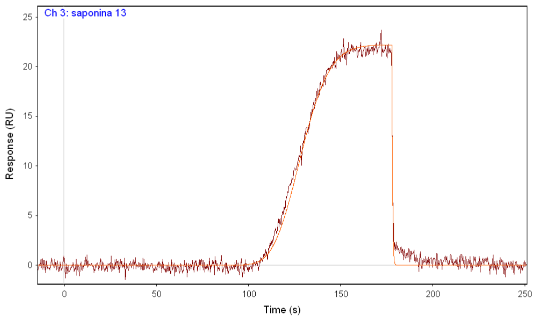
Ch 3: saponina 13
Response (RU)
Time (s)

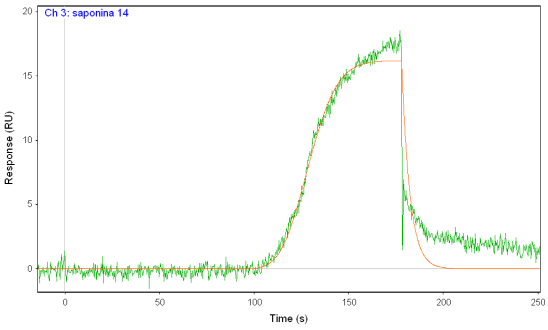
Ch 3: saponina 14
Response (RU)
Time (s)

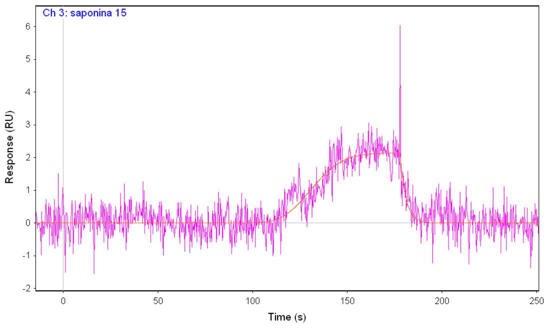
Ch 3: saponina 15
Response (RU)
Time (s)

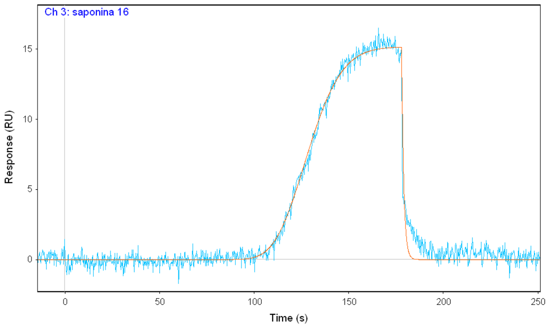
Ch 3: saponina 16
Response (RU)
Time (s)

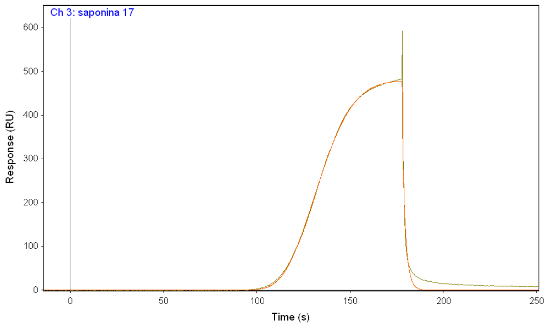
Ch 3: saponina 17
Response (RU)
Time (s)

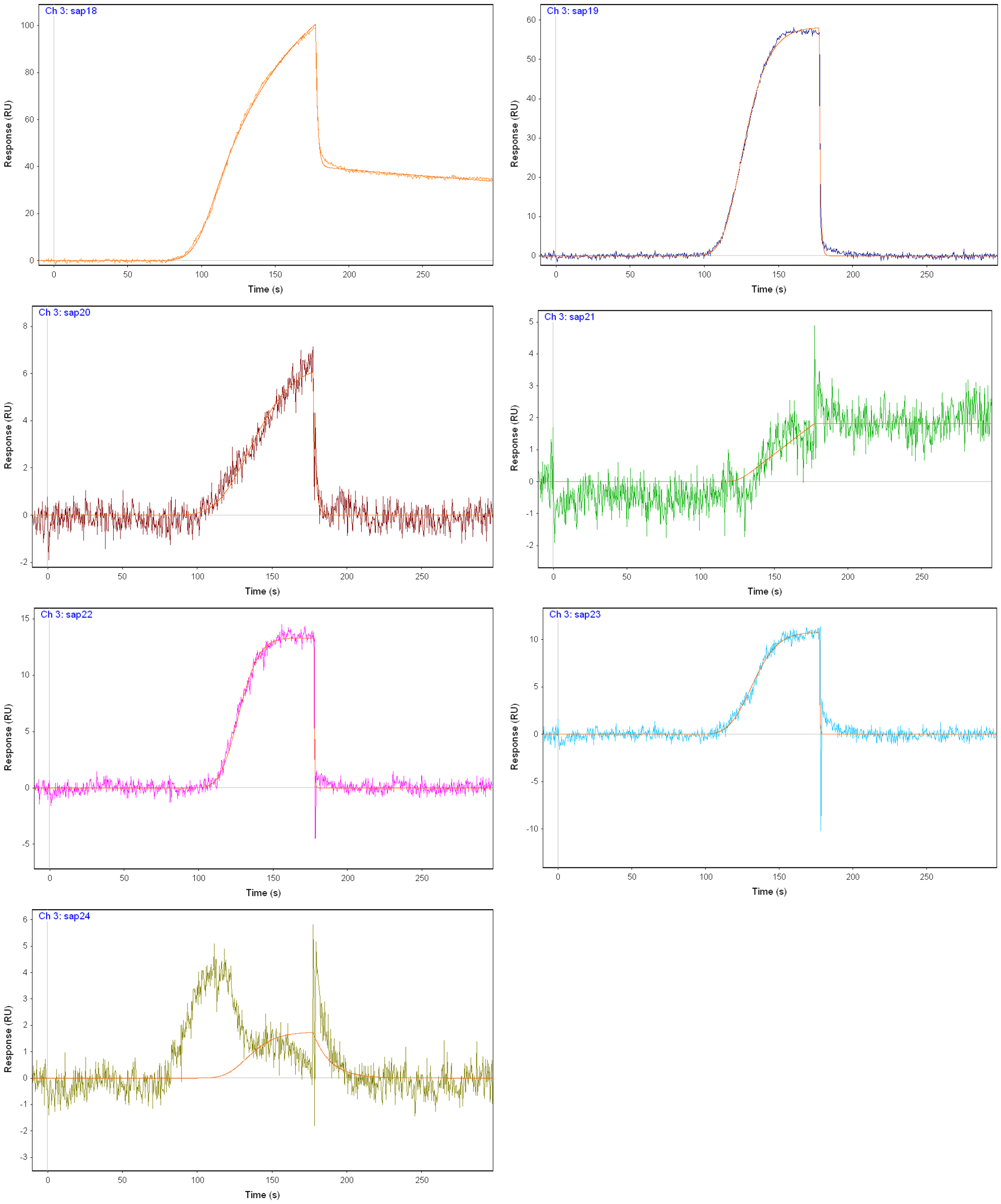

Supplementary Figure S1: Representative data sets for kinetic analysis of saponin/sapogenin-PPARγ interactions. Red lines represent the global fits of the data to a 1:1 bimolecular interaction model (with the exception of **LT175**, **17** and **18** for which a 2:1 model gave a better fit). The data of saponine **12**, **21** and **24** could not be fitted to the interaction models. The kinetic parameters obtained from each interaction are reported in Table 2.

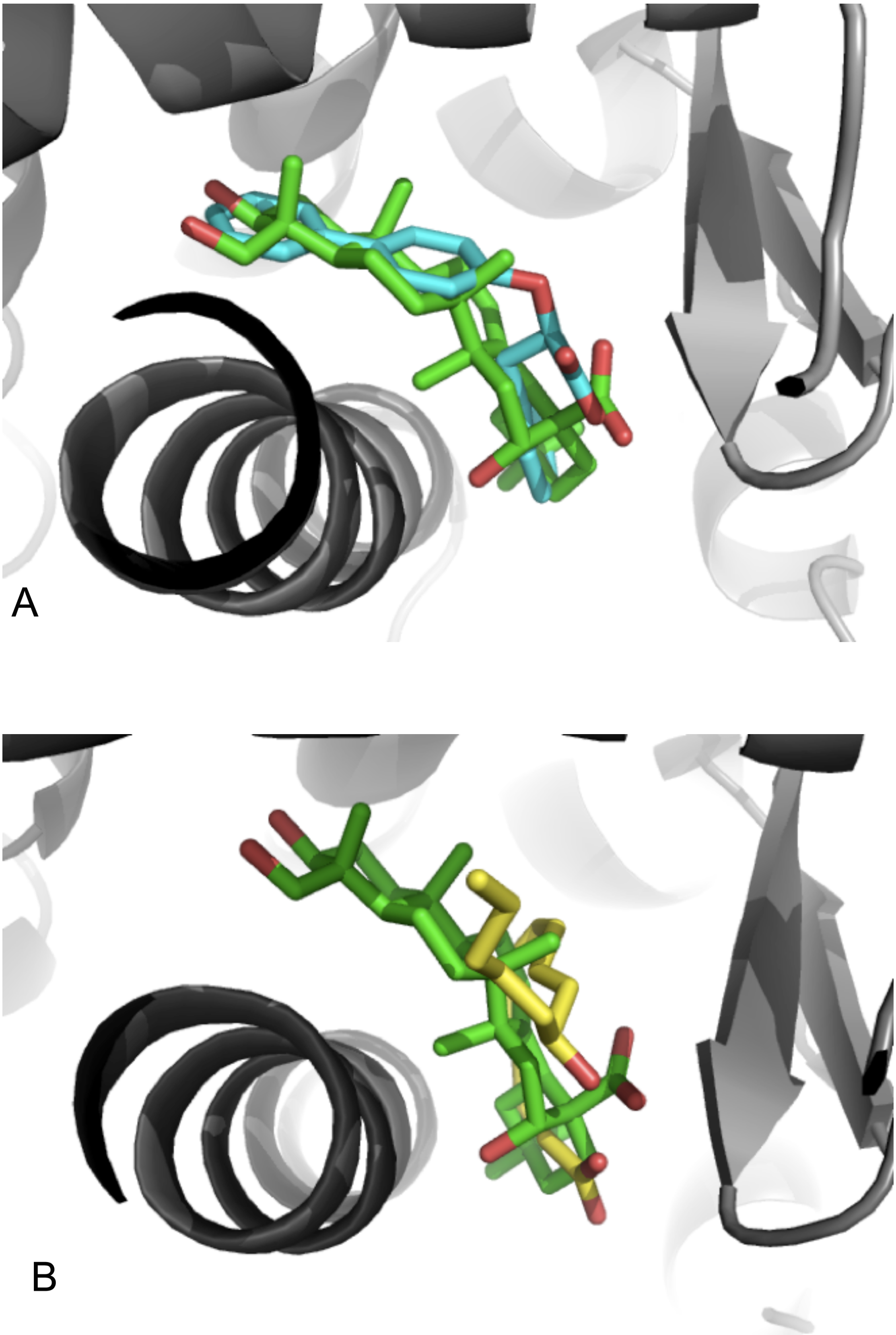
A
B

Supplementary Figure S2: Superposition of (A) **19** (green) and LT175R (cyan) (pdb code 3D6D) and (B) **19** (green) and 13-(*S*)-HODE (yellow) (pdb code 2VST), in the LBD of PPARγ.

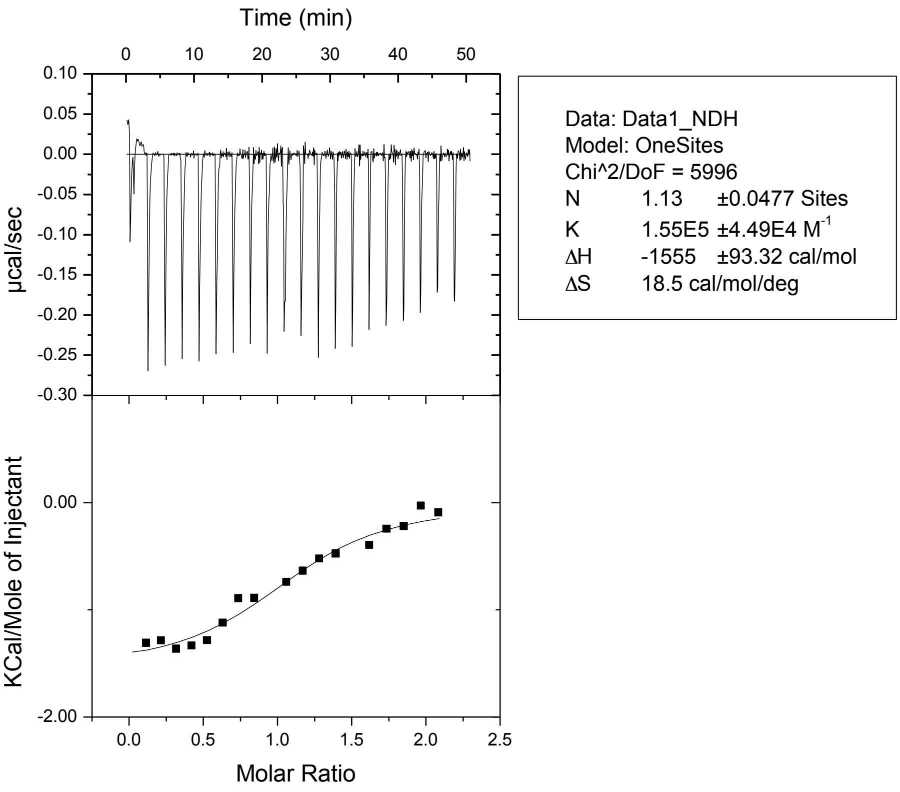

Fig. S2(A): SAP19 (500 μM), PPARγ (50 μM)

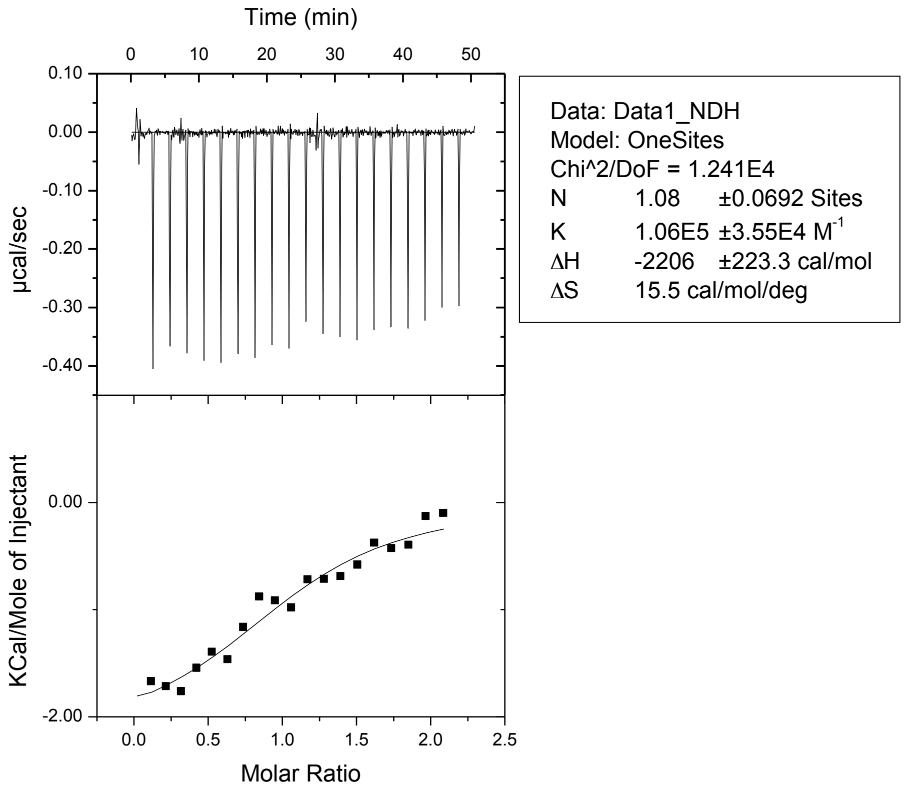

Fig. S2(B): 13-(*S*)-HODE (400 μM), PPARγ (50 μM)

Supplementary Figure S3: Titration of (A) **19** and (B) 13-(*S*)-HODE to PPARγ-LBD. The upper panel of each figure shows the raw data of the ITC experiment. The lower panel shows the corresponding binding isotherm fitted according to the "one binding site" model.
